# Bio-Inspired Feedback Visual Network for Robust Small-Target Motion Detection in Complex Environments

**DOI:** 10.3390/biomimetics11030188

**Published:** 2026-03-04

**Authors:** Jun Ling, Jing Yao, Botao Luo, Wenli Huang

**Affiliations:** 1 The School of Mathematics and Computational Science, Shangrao Normal College, Shangrao 334001, China; 305199@sru.edu.cn (J.L.); 305200@sru.edu.cn (J.Y.); 23010320@sru.edu.cn (B.L.); 2 Postdoctoral Research Workstation of Mltor Numerical Control Technology Limited Company, Zhongshan 528400, China

**Keywords:** small object motion detection, feedback mechanism, response range, insect visual system

## Abstract

In dynamic and complex real-world environments, artificial intelligence (AI) vision systems continue to face significant challenges in accurately detecting and tracking small objects. The core difficulty lies in the fact that small targets usually exhibit limited spatial and textural features, while dynamic backgrounds often generate numerous misleading motion cues, thereby interfering with reliable discrimination between targets and backgrounds. Inspired by the remarkable capability of the insect brain in detecting small moving objects, this study proposes a visual neural network model enhanced by a feedback mechanism. By adaptively responding to temporal variations, the proposed model is able to more precisely distinguish small targets from background-induced false targets. The network architecture consists of two main pathways: a motion detection pathway that extracts motion-related features from minute targets, and a feedback attention pathway that enhances the focus on true targets by leveraging the feature differences between real and false motion signals. In addition, a global inhibition module is incorporated to reduce the false alarm rate by filtering out background-induced false positives, thereby improving the overall detection performance of the model. Experimental results demonstrate that the proposed model achieves a detection rate of 0.81 in complex visual scenarios, whereas the compared models all achieve detection rates below 0.59, indicating a significant improvement in detection performance. Meanwhile, in terms of Precision and F1-score, the proposed model achieves values of 0.0648 and 0.12, respectively, while the compared models obtain values lower than 0.0077 and 0.015, further validating the superiority of the proposed method in detection accuracy and robustness.

## 1. Introduction

### 1.1. Background

**Figure 1:**
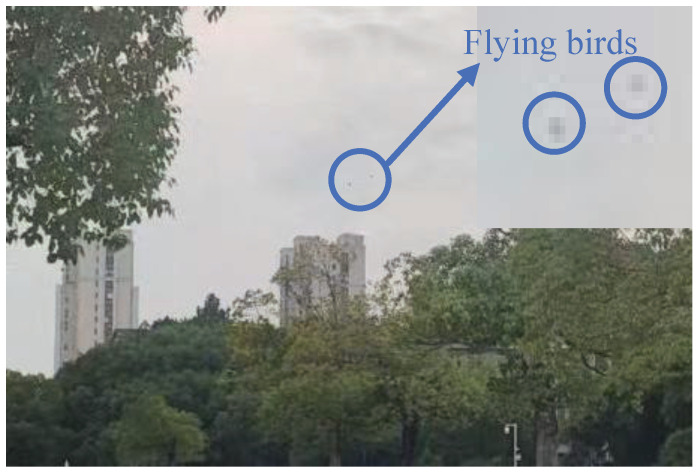
Small objects in a natural environment. Source: author’s contribution.

Tracking of moving targets is a core component of computer vision, where it has been extensively applied in diverse practical domains, including surveillance systems [1], autonomous systems [2], and early warning technologies [3]. However, accurately tracking small objects in complex and dynamic environments remains a critical and unresolved challenge. This problem primarily arises from two aspects. First, small targets—especially those located far from the camera—generally suffer from limited spatial resolution and insufficient discriminative features. They are often tiny and lack distinctive visual characteristics (as shown in Figure 1), making the extraction of meaningful features particularly difficult. Second, in natural scenes, tiny targets are often hidden within complex backgrounds, making them difficult to differentiate from visually similar elements, such as flowers, shadows, or stones. Consequently, developing a robust visual system capable of reliably detecting and interpreting the motion of small objects in complex environments has become a crucial research direction in computer vision.

Biological investigations have revealed that specific insects, such as dragonflies, exhibit remarkable visual capabilities, enabling them to efficiently track small flying targets even in complex environments, with pursuit success rates reaching up to 97% [4]. The underlying mechanism of this ability is mainly linked to a specialized neuronal class in the insect brain referred to as Small-Target Motion Detectors (STMDs). Research indicates that STMD neurons are highly tuned to small moving targets spanning a visual angle of approximately 1°–3°, but respond only weakly, or not at all, to larger stimuli above 10° [5,6]. Further experimental evidence indicates that these neurons remain effective in detecting and responding to small moving targets even against complex and dynamic backgrounds, demonstrating strong anti-interference capability and selectivity. Thus, the remarkable performance of the insect visual system in sensing and following small targets offers important biological understanding and guides the development of bio-inspired visual neural architectures.

With increasing insight into the electrophysiology of STMD neurons, various visual networks have been proposed to enable efficient detection of small-target motion in cluttered environments. Early models, such as Elementary Small-Target Motion Detector (ESTMD) and direction detection visual network (DSTMD) [7,8], successfully simulated size-selective and direction-selective responses by correlating successive luminance changes or integrating spatial information. However, these initial frameworks soon revealed a critical limitation: their performance in accurately detecting small targets in complex and dynamic backgrounds was relatively low. The main reason is that background features similar to the target can easily cause false detections, thereby reducing detection accuracy. Further, to detect small targets in low-sampling-rate videos, Xu et al. introduced the fractional-order visual network (Frac-STMD) [9]; meanwhile, Wang et al. integrated attention mechanisms into the ESTMD framework to develop the attention-based apg-STMD model (apg-STMD) [10]. This approach leverages both prediction and attention mechanisms to dynamically refine detection results from previous frames, boosting the performance in detecting tiny moving objects.

Feedback, as a fundamental regulatory mechanism, is widely observed across diverse biological taxa and serves to optimize system stability and efficiency through information loops [11,12,13]. In biological neural systems, a representative example is the midbrain circuitry of weakly electric fish (Apteronotus leptorhynchus), in which feedback interactions between the torus semicircularis and the superficial layers of the electrosensory lateral line lobe (ELL) balance excitation and inhibition, suppress global clutter while synthesizing motion representations. Similar top-down inhibitory modulation has also been reported in the mammalian cortico-geniculate pathway (V1 → LGN) as well as in the centrifugal fibers of the avian retina. In insects, although recent studies have identified potential feedback candidate neurons in the optic lobe, such as medulla feedback neurons (MF1–MF4), their precise functional roles in small-target motion detection remain largely unexplored. We emphasize that the proposed feedback mechanism is functionally inspired by these biological principles rather than representing a strict anatomical replication of a specific insect neural circuit. Motivated by this biologically grounded concept of feedback gating, this paper proposes a novel visual neural network termed RFSTMD. The proposed model integrates two interacting pathways: a feedforward detection pathway for extracting spatiotemporal motion features, and a top-down feedback pathway that selectively enhances responses at true small-target locations. To systematically evaluate the proposed framework, this research is predicated on two core hypotheses:

**Hypothesis 1.** 
*The feedback modulation pathway effectively differentiates small moving targets from background clutter by utilizing temporal difference features.*

**Hypothesis 2.** 
*The synergy between the feedback pathway and global inhibitory mechanism significantly enhances detection performance compared to traditional feed-forward STMD models.*

The following sections detail the materials, algorithmic implementation, and experimental setup used to test these hypotheses.

### 1.2. Novelty of the RFSTMD Model

Although previous research has explored the application of feedback mechanisms in small-target detection (e.g., temporal feedback model (Feedback STMD) [14], the delayed feedback network (FSTMD) [15] and the spatio-temporal feedback model (ST-STMD) [16]), the RFSTMD model introduces key innovations in both structural design and discrimination logic. Traditional delayed-feedback models typically leverage temporal persistence through amplitude- or accumulation-based gain modulation. However, in dynamic backgrounds with strong temporal correlations, such mechanisms are prone to “false reinforcement,” leading to elevated false alarm rates.

In contrast, RFSTMD incorporates a response-range-guided feedback gating mechanism. Specifically, the feedback signal is modulated by the temporal variation of neural responses rather than by their absolute amplitude. This design shifts the feedback paradigm from a “state-driven” to a “trend-driven” approach, making the system more sensitive to the transient features of small targets.

In conventional frameworks, sustained responses are often interpreted as indicators of true targets. RFSTMD, however, recognizes that real small targets induce pronounced transient fluctuations, whereas background interference signals tend to remain relatively smooth or low-frequency. By integrating the temporal response range into the feedback loop, the system selectively amplifies target-induced responses, thereby significantly enhancing attention to relevant stimuli.

Furthermore, this mechanism offers substantial computational advantages. Temporal variability can be efficiently approximated using simple difference operations (Max–Min), avoiding complex energy integration or higher-order statistical computations. This efficient design not only improves detection performance but also minimizes computational overhead, making RFSTMD highly suitable for real-time deployment on resource-constrained neuromorphic chips or edge computing platforms.

In summary, RFSTMD emphasizes temporal non-stationarity rather than average persistence, enhancing robustness against oscillatory background noise while providing an efficient and interpretable computational solution. The model integrates a feedforward pathway for extracting spatiotemporal motion features with a top-down feedback pathway that selectively enhances target responses. Through the synergistic optimization of these two pathways, RFSTMD offers a biologically inspired and reliable solution for real-time small-target detection in cluttered environments.

### 1.3. Literature Review

This subsection primarily reviews existing research on insect-inspired motion perception neural networks and various approaches to infrared tiny object detection. In addition, it outlines related studies on feedback regulation.

#### 1.3.1. Bio-Inspired Visual Network Modeled on Insect Vision

Physiological studies have revealed that insects possess the ability to perceive a diverse range of motion types within their visual fields, including cues indicating imminent collisions, large-field movements, and the motion of small targets. This advanced motion perception is primarily facilitated by three distinct neuron classes. Lobula giant movement detectors (LGMDs) [17,18] exhibit strong sensitivity to approaching stimuli, playing a pivotal role in collision avoidance. In contrast, lobula plate tangential cells (LPTCs) [19,20] respond preferentially to broad-field motion, supporting flight stabilization and navigational control. Small target motion detector (STMD) neurons [21] are specifically tuned to detect minute moving objects against visually cluttered backgrounds. Together, these neuron types constitute a coordinated network that enables insects to efficiently perceive and react to dynamic visual scenes. LGMD neurons display directional selectivity, responding robustly to looming stimuli while showing markedly reduced activity for receding motion. This characteristic has inspired numerous bio-inspired visual network models aimed at collision detection [22,23,24], which leverage the LGMD’s selective sensitivity to enhance detection of approaching objects while suppressing false positives from background motion. Such models have demonstrated the ability to replicate insect-like collision avoidance behaviors in complex and rapidly changing environments. By comparison, LPTC neurons are tuned to wide-field motion, particularly when stimuli occupy a large portion of the visual scene. Drawing on these functional properties, computational models—such as the expansion-motion detector (EMD) [25], two-quadrant detector (TQD) [26], and weighted-quadrant detector [27]—have been developed to process global motion cues efficiently. These approaches emulate the LPTC’s capacity to integrate motion information across the entire visual field, thereby providing robust recognition of large-scale movement in cluttered or dynamic environments. Despite their success in replicating collision avoidance and wide-field motion detection, these bio-inspired models remain limited in spatial resolution, constraining their ability to accurately detect or distinguish small objects.

STMD neurons are inherently specialized for detecting motion from small targets, exhibiting high sensitivity even when such targets are embedded within visually cluttered or dynamic backgrounds. Building on this intrinsic selectivity, researchers have developed a series of STMD-inspired visual networks designed to simulate and extend these capabilities in computational models. These include ESTMD [7] and DSTMD [8], which focus on feature extraction and directional selectivity; the temporal feedback model (Feedback STMD) [14] and the delayed feedback network (FSTMD) [15], which introduce temporal recurrence to enhance motion tracking across consecutive frames; the spatio-temporal feedback model (ST-STMD) [16], which integrates spatial and temporal information for more robust detection; as well as the apg-STMD model [10] and fractional-order STMD [9], which further optimize small-target detection through advanced computational strategies. Collectively, these models have been shown to reliably detect and track small moving objects within complex and dynamic visual environments, highlighting the potential of biologically inspired neural mechanisms in addressing challenging visual tasks. Despite their promising performance in dynamic visual scenes, accurately discriminating small targets from background clutter under densely cluttered conditions remains challenging, underscoring the need for more robust and adaptive small-target detection mechanisms.

#### 1.3.2. The Role of Feedback Regulation

Feedback serves as a fundamental regulatory mechanism widely observed in natural systems. It operates through information loops that continuously adjust a system’s dynamics, thereby optimizing its overall performance. Extensive theoretical and empirical studies have shown that integrating feedback regulation can substantially improve a system’s stability, adaptability, and efficiency. For example, Ren et al. [11] proposes a multi-level feedback feature extractor that dynamically optimizes feature selection through image-level and instance-level feedback, enabling adaptation to variations in image quality and classification uncertainty. Experiments demonstrate that Flex markedly boosts detection accuracy on several aerial object datasets (DOTA, HRSC) and the general MSCOCO dataset, highlighting the feedback mechanism’s effectiveness and versatility in object detection. Broad et al. [12] proposes a bio-inspired image recognition model that integrates feedforward and feedback processes, using saccade-like attention focusing to enhance the accuracy and interpretability of large-scale image classification. Xu et al. [13] proposes a dynamic vehicle tracking framework based on LiDAR, which achieves accurate pose estimation and real-time tracking under sparse or incomplete point clouds by leveraging motion feedback, a heading-normalized vehicle model, and the Interactive Multiple Model (IMM) approach.

During the last decade, researchers have applied feedback mechanisms broadly in nonlinear dynamic systems to ensure stability and to create robust controllers [28,29,30,31,32,33]. Despite their demonstrated effectiveness across diverse domains, the modeling and functional roles of feedback within STMD neural pathways remain poorly understood. Therefore, investigating feedback processes in STMD pathways, along with the associated information processing mechanisms, is essential for deepening our understanding of visual information regulation in biological systems and for guiding the development of neuromorphic computing architectures inspired by such biological principles.

#### 1.3.3. Small Infrared Target Detection

Small infrared target detection is primarily concerned with identifying weak thermal signatures emitted by objects such as missiles, bombs, and other high-value targets. Detecting these small targets is inherently challenging due to low contrast, complex backgrounds, and noise interference. Over the past decade, numerous algorithms have been proposed to address these difficulties and improve detection performance. For instance, Meng et al. [34] proposed the Multiple Information and Noise Predictions Network (MINP-Net), a robust infrared small-target detection framework that combines multiscale feature extraction, noise prediction, and regional localization. In addition, they introduced the large-scale NCHU-Seg dataset, which provides extensive annotated examples for training and evaluation. MINP-Net achieves superior detection accuracy while maintaining balanced false alarm rates compared with state-of-the-art methods, demonstrating its effectiveness in handling complex infrared scenes. Similarly, Liu et al. [35] developed a Scale and Location-Sensitive (SLS) loss function in combination with a straightforward Multi-Scale Head Network (MSHNet), explicitly designed to enhance the detection of small infrared targets. This approach improves both localization precision and scale awareness, enabling the model to more reliably identify targets of varying sizes and positions. Experimental results show that MSHNet significantly outperforms existing state-of-the-art techniques in terms of both accuracy and robustness, highlighting the advantages of incorporating scale and location sensitivity into infrared small-target detection frameworks. Yan et al. [36] proposes a learning-based Spatio-Temporal Differential Multiscale Attention Network (STDMANet), which achieves high-precision detection of infrared dim small targets under complex backgrounds by integrating spatio-temporal multiscale features and introducing a mask-weighted heatmap loss, significantly outperforming existing methods. In addition, researchers have proposed various methods [37,38,39] to achieve effective detection of infrared small-target motion under different scenarios. While capable of detecting small-target motion, these approaches are highly sensitive to thermal differences and depend on clear backgrounds, conditions that are uncommon in real-world settings.

The structure of this paper is as follows. Section 2 (Materials and Methods) details the proposed RFSTMD model, along with the experimental setup and parameter configurations. Section 3 (Results) presents the results, including comparative analyses and evaluations of robustness under extreme conditions. Section 4 (Discussion) provides further discussion and interpretation of the findings. Finally, Section 5 (Conclusions) summarizes the work and highlights directions for future research.

## 2. Materials and Methods

This section presents the mathematical formulation of the proposed model and introduces the datasets used to validate its effectiveness, the experimental parameter settings, and the detailed experimental procedures.

### 2.1. Model Architecture

**Figure 2:**
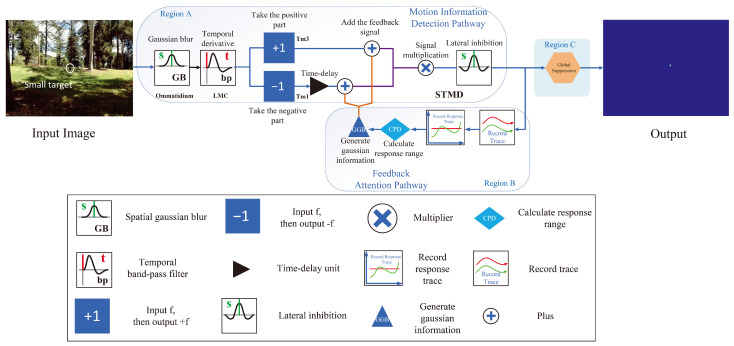
Flowchart of the proposed RFSTMD model. The signal originates from Region A, propagates through the feedback loop between Region A and Region B, and is finally integrated into Region C for output. Source: Author’s contribution.

The realization process of the RFSTMD model is illustrated through the self-explanatory flowchart in Figure 2, which is categorized into three functional segments: Region A (Motion detection pathway), Region B (Feedback attention pathway), and Region C (Global suppression). As indicated by the signal flow, the processing pipeline originates in Region A, where external visual signals are captured by the ommatidia and pre-processed by Large Monopolar Cells (LMCs) to extract instantaneous luminance variations. These signals are subsequently transmitted to Tm3 and Tm1 neurons, converging to produce an initial response in the STMD neurons. For instance, in the task of detecting small flying targets, such as birds or drones against cluttered backgrounds, this initial response identifies potential target locations based on their spatiotemporal motion cues. Following this, the information enters Region B, where signals are synergistically processed through a loop constructed by the feedback attention mechanism with the Tm3 and Tm1 signals of the subsequent time step. This allows the system to selectively enhance target-related cues across successive time steps. Finally, the modulated information propagates to Region C, where a global inhibition mechanism is applied to suppress background clutter, resulting in a precise motion response with a high signal-to-noise ratio at the STMD neurons. By structuring the model into these explicit logical areas (Region A → Region B → Region C), the complex neural integration process becomes intuitive and verifiable.

**Figure 3:**
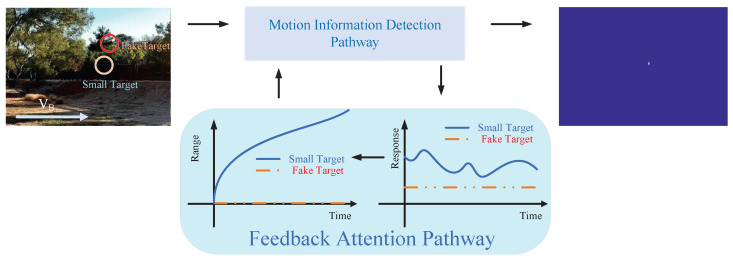
The The working mechanism of feedback attention pathway. The short arrows denote the direction of information flow, while the long arrows represent the coordinate axes. Source: author’s contribution.

The feedback pathway of Region B is designed based on the distinct temporal behavior of true targets versus background-induced fake features. As shown in Figure 3, when a small target moves relative to the background, its response exhibits significant fluctuations over time *t*. In contrast, fake features, which remain relatively stationary with respect to the moving background, show minimal temporal variation. The magnitude of response fluctuations, quantified as the range over time, serves as a feedback signal and provides a reliable criterion for identifying small-target positions.

#### 2.1.1. Motion Information Detection Pathway

The motion information detection pathway is largely built upon a fundamental STMD visual neural network, which emulates the hierarchical processing observed in insect vision. Initially, the ommatidia capture luminance signals from the visual scene, providing the raw input for subsequent processing. These signals are then transmitted to LMC neurons, which extract temporal changes in luminance. The processed signals are then relayed to Tm3 and Tm1 neurons, where parallel processing further refines motion features. Finally, STMD neurons integrate the outputs of both pathways, enabling the network to detect and respond to small moving targets with high precision, even under complex visual conditions. This sequential processing ensures that subtle motion cues are effectively amplified and combined, forming the core mechanism underlying small-target motion detection in the STMD network.

The ommatidia act as the initial signal receptors in the motion information detection pathway (Figure 2) [40,41], capturing visual brightness stimuli from the real environment. These receptors employ Gaussian filters for inherent Gaussian blurring during processing. We denote the brightness received by ommatidia neurons mathematically as I(x,y,t)∈R. The resulting ommatidia response, P(x,y,t), is defined as follows:

(1) $$P\left(x,y,t\right)=\int \int I\left(u,v,t\right)G_{\sigma_{1}}\left(x-u,y-v\right)dudv,$$

here, $G_{\sigma_{1}}\left(x,y\right)$ denotes the Gaussian kernel function:

(2) $$G_{\sigma_{1}}\left(x,y\right)=\frac{1}{2\pi \sigma_{1}^{2}}e^{\frac{-(x^{2}+y^{2})}{2\sigma_{1}^{2}}},$$

$\sigma_{1}$ denotes the standard deviation parameter of the Gaussian function.

Figure 2 illustrates the Large Monopolar Cells, which are postsynaptic to the ommatidia and are highly sensitive to temporal variations in luminance, enabling effective detection of motion or luminance changes [26,27]. In biological vision, LMCs emphasize such temporal changes to suppress redundant static information. Within the proposed model, the LMC layer is designed as a temporal band-pass filter to capture luminance variations from the ommatidia outputs. Computationally, this temporal band-pass filtering is simulated by convolving the ommatidia outputs with two temporal kernels, thereby approximating the LMCs’ response to luminance changes. The LMC response is formulated as:

(3) L(x,y,t)=∫P(x,y,s),H(t−s),ds,

where L(x,y,t) denotes the output of the LMC at spatial location (x,y) and time *t*, and P(x,y,t) represents the corresponding ommatidia output. The kernel H(t) for temporal filtering is obtained by computing the difference between a pair of Gamma kernels.

(4) $$H\left(t\right)=\Gamma_{n_{1},\tau_{1}}\left(t\right)-\Gamma_{n_{2},\tau_{2}}\left(t\right),$$

with

(5) $$\begin{array}{c} \Gamma_{n,\tau }\left(t\right)=\left\{\begin{array}{cc} {\left(nt\right)}^{n}\frac{e^{\frac{-nt}{\tau }}}{\left(n-1\right)!\cdot \tau^{n+1}}, & \mathrm{for}\;\;t\geq 0,\\ 0, & \mathrm{for}\;\;t<0. \end{array}\right. \end{array}$$

Here, *n* and τ denote the order and temporal constant of the Gamma kernel, respectively [42]. The temporal delay and shape of the Gamma kernel can be readily controlled by varying the time constant *n* and order τ. Simultaneously, the LMC’s delay is configured to fully capture the luminance changes at each pixel caused by small target motion.

Figure 2 illustrates the Tm3 and Tm1 neurons, which are postsynaptic cells to the LMCs and located in the medulla of the insect visual system. These neurons act as relays, transmitting information from the lamina to higher-order visual centers. They primarily receive input from the LMCs and project processed signals further along the visual pathway. Tm3 is tuned to detect luminance increments, whereas Tm1 detects luminance decrements, with a slight temporal delay relative to Tm3 [43]. Functionally, Tm3 and Tm1 are analogous to half-wave rectifiers, separating ON (luminance increase) and OFF (luminance decrease) signals to enable parallel processing of motion information. Computationally, this half-wave rectification is simulated by extracting the positive and negative components of the LMC outputs. The output of the Tm3 neuron $S_{ON}\left(x,y,t\right)$, is obtained by extracting the positive component of L(x,y,t):

(6) $$S_{ON}\left(x,y,t\right)={\left[L(x,y,t)\right]}^{+}.$$

Previous studies have shown that Tm1 exhibits a temporal lag relative to Tm3 at the same spatial positions (x,y). According to the proposed framework, $S_{OFF}\left(x,y,t\right)$, representing Tm1 activity, is defined as follows:

(7) $$S_{OFF}\left(x,y,t\right)=\int {\left[L\left(x,y,s\right)\right]}^{-}\Gamma_{n_{3},\tau_{3}}\left(t-s\right)ds,$$

here, ${\left[x\right]}^{+}$ and ${\left[x\right]}^{-}$ are defined as the positive and negative parts of *x*, i.e., max(x,0) and min(x,0). $n_{3}$ and $\tau_{3}$ denote the order and temporal constant of the $\Gamma_{n_{3},\tau_{3}}$, respectively. The time-delay parameter $\tau_{3}$ is configured to match the duration for a small target to traverse a single pixel, ensuring temporal alignment between the outputs of Tm3 and Tm1.

Furthermore, prior to the correlation of the positive and negative signals, the outputs of the Tm3 and Tm1 neurons must be updated by introducing a feedback signal F(x,y,t) from the previous time step. Let F(x,y,t) denote the feedback signal. The resulting new positive and negative signals are defined as:

(8) $$S_{ON}^{\prime }\left(x,y,t\right)=S_{ON}\left(x,y,t\right)+F\left(x,y,t\right),$$

(9) $$S_{OFF}^{\prime }\left(x,y,t\right)=S_{OFF}\left(x,y,t\right)+F\left(x,y,t\right).$$

STMD neurons are located in the higher layers of the insect visual system, and are highly sensitive to the motion of small targets, responding reliably even in complex backgrounds or under motion interference [8,12,44]. They are specialized for detecting small-target motion and function as motion-sensitive detectors within the network, receiving inputs from Tm1 and Tm3. Through nonlinear integration of Tm1 and Tm3 outputs, STMD neurons produce pronounced responses to small-target motion while suppressing interference from larger objects, thereby enabling effective detection of small targets. In the computational implementation, the STMD output at position (x,y) is obtained by the nonlinear multiplication of $S_{ON}^{\prime }\left(x,y,t\right)$ and $S_{OFF}^{\prime }\left(x,y,t\right)$, specifically expressed as:

(10) $$S\left(x,y,t\right)=S_{ON}^{\prime }\left(x,y,t\right)\times S_{OFF}^{\prime }\left(x,y,t\right).$$

Furthermore, a lateral inhibition mechanism is applied to the STMD output S(x,y,t) to filter out large objects (see Figure 1). Thus:

(11) $$D\left(x,y,t\right)={\left[\int \int \;S\left(u,v,t\right)K\left(x-u,y-v\right)dudv\right]}^{+},$$

here, K(x,y) denotes the inhibition kernel, i.e.,

(12) $$\begin{array}{c} K\left(x,y\right)=\varphi {\left[g\left(x,y\right)\right]}^{+}+\psi {\left[g\left(x,y\right)\right]}^{-}, \end{array}$$

g(x,y), constructed by combining two Gaussian functions linearly and subtracting a fixed constant, is given by:

(13) $$\begin{array}{c} g\left(x,y\right)=G_{\sigma_{2}}\left(x,y\right)-eG_{\sigma_{3}}\left(x,y\right)-\rho . \end{array}$$

The symbols φ,ψ,e,ρ represent fixed constants in the equation, and their values depend on the model’s preference for target size.

#### 2.1.2. Feedback Attention Pathway

This subsection presents a mathematical representation of the feedback attention pathway.

**Figure 4:**
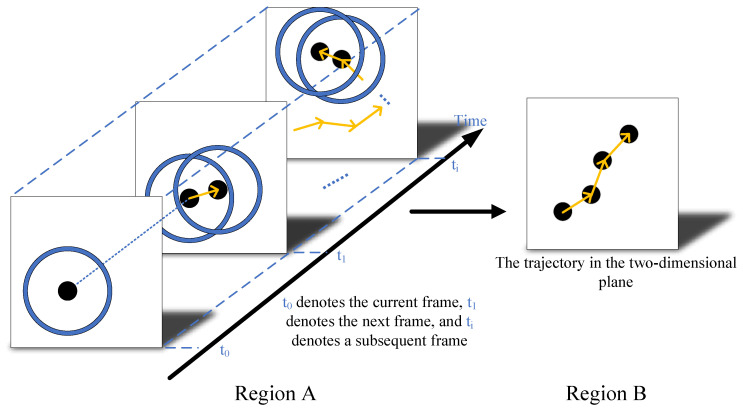
The region A illustrates the method for tracking the motion trajectories of small features. The region B presents the recorded target trajectories projected onto a 2D plane. The blue circles represent the spatial region centered on the black small targets, the long black arrow indicates the time axis, the short black arrow indicates the mapping process, and the orange arrows indicate the motion direction of the targets.

As illustrated in Figure 4, RFSTMD employs the motion information detection pathway to both identify target positions and capture the complete motion trajectories of small features. The position of the feature is identified based on a detection threshold ϑ (5 pixels). At a fixed time $t_{0}$, a small feature is tracked at location $\left(x_{0},y_{0}\right)$ if the module output $D(x_{0},y_{0},t_{0})$ satisfies $D(x_{0},y_{0},t_{0})>\vartheta$. Similarly, the location $\left(x_{1},y_{1}\right)$ is tracked and recorded at the next time point $t_{1}$. We determine that $\left(x_{1},y_{1}\right)$ at $t_{1}$ and $\left(x_{0},y_{0}\right)$ at $t_{0}$ belong to the same trajectory if $\left(x_{1},y_{1}\right)$ falls within a small spatial neighborhood of $\left(x_{0},y_{0}\right)$. By repeating this process, a motion trajectory MT can be obtained over a period of time. To account for occlusions or trajectory intersections, if a target temporarily disappears due to being occluded or overlapping with another trajectory, the system delays the termination of the original trajectory and continues it once the target reappears. If the target is completely lost, a new trajectory is automatically initiated. This ensures that the motion trace MT captures the feature’s trajectory as completely as possible, even under temporary interruptions. In this framework, the motion trace MT is defined as:

(14) $$\begin{array}{c} MT=\left(x\left(t\right),y\left(t\right)\right),t\in \left[t_{begin},t_{current}\right]. \end{array}$$

Here, (x(t),y(t)) represent the pixel positions at time *t*, while $t_{begin}$ and $t_{current}$ denote the starting and current time points.

For each MT, the associated response trace $D_{RC}$ is derived from the feature location and its corresponding response output, and is defined as follows:

(15) $$\begin{array}{ccc} D_{RC} & =(D(x(t)),D(y(t))), & \;\;\;\;\;\;\;\;\;\;\;\;\;\;\;t\in [t_{begin}.t_{current}]. \end{array}$$

**Figure 5:**
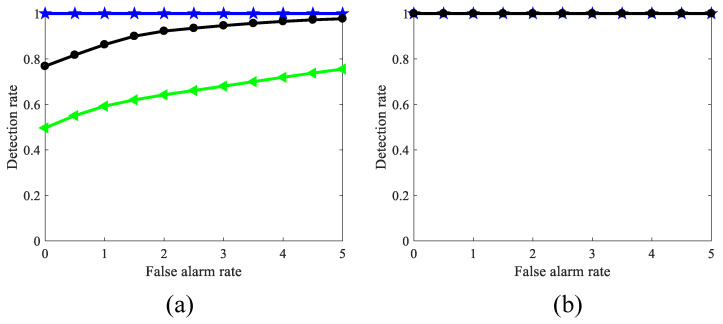
(**a**) Comparison of detection performance using different temporal discriminative features. Response range (Blue), response gradient (Black), and temporal contrast (Green). (**b**) Comparison of detection performance using different temporal statistics features. Response range (Blue), standard deviation (Black). Source: author’s contribution.

After obtaining $D_{RC}$, accurately distinguishing true small targets from background-induced false positives becomes crucial. Existing temporal discriminative methods primarily rely on temporal contrast or gradient features, but extracting these features typically incurs relatively high computational costs (see Table 1). In contrast, the proposed response range, as a temporal feature, not only maintains low computational complexity but also demonstrates superior detection performance compared to conventional features (see Figure 5). Although ablation experiments indicate that, on general computing platforms, the response range and standard deviation achieve comparable detection performance and per-frame runtime, we adopt the response range as the primary criterion for target discrimination due to its advantages in both hardware efficiency and alignment with biological mechanisms. In terms of hardware implementation, standard deviation involves computationally expensive operations such as squaring, summation, and square roots, whereas the response range (Max–Min) relies solely on basic comparison operations, making RFSTMD significantly more efficient for deployment on low-power neuromorphic chips or FPGA-based vision sensors. Biologically, the response range more directly captures the characteristic pulse-like peak responses of STMD neurons. Compared to time-averaged statistical measures that smooth the signal (e.g., standard deviation), it is more sensitive to transient contrast changes, effectively highlighting signals induced by small targets while suppressing relatively stable background noise. Although there is no direct evidence that insect neurons explicitly compute a temporal response range, this mechanism serves as a bio-inspired engineering approximation: it efficiently captures temporal consistency to discriminate true targets from background clutter without literally replicating neural computations, thereby enabling precise and selective enhancement of micro-moving targets in complex environments.

For each response trajectory, the response range $R_{V}$ is evaluated at every time step *t* and is defined as follows:

(16) $$\begin{array}{ccc} R_{V} & =(D_{RC}\left(x\left(t\right)\right),D_{RC}\left(y\left(t\right)\right),t),\;\;\;\;\;\; & t\in [t_{begin},t_{current}]. \end{array}$$

To accurately identify small targets, the range $R_{V}$ is compared against a predefined threshold υ. A small target is deemed present at pixel $\left(x_{0},y_{0}\right)$ and time $t_{0}$ if $R_{V}\left(x_{0},y_{0},t_{0}\right)$ exceeds υ. Setting the threshold too high may result in true small targets being misclassified as false positives, while a threshold that is too low can increase the likelihood of missed detections. Therefore, the threshold must be carefully selected based on the sensitivity of detection performance (see the Parameter Settings subsection) to ensure an appropriate balance between false alarms and missed targets.

The system finally generates a feedback signal based on the detected target position. Specifically, F(x,y,t) is computed by applying a spatial Gaussian filter G(·) to the detection response D(x,y,t), centered at $\left(x_{T},y_{T}\right)$, to amplify the response in that area. The feedback signal is given by:

(17) $$F\left(x,y,t\right)=a\frac{1}{2\pi \sigma^{2}}\exp\left(-\frac{{\left(x-x_{T}\right)}^{2}+{\left(y-y_{T}\right)}^{2}}{2\sigma^{2}}\right)$$

where *a* is feedback coefficient and σ is a fixed standard deviation that determines the spatial spread of the Gaussian function.

#### 2.1.3. Global Suppression

To eliminate background false positives, the model ultimately employs a global suppression mechanism. Specifically, the suppressed feature map can be expressed as

(18) $$F_{\mathrm{suppressed}}\left(x,y,t\right)=F\left(x,y,t\right)-\lambda \;B\left(x,y,t\right)$$

where

(19) $$B\left(x,y,t\right)=\int \int F\left(u,v,t\right)G_{\sigma_{4}}\left(x-u,y-v\right)dudv,$$

**Figure 6:**
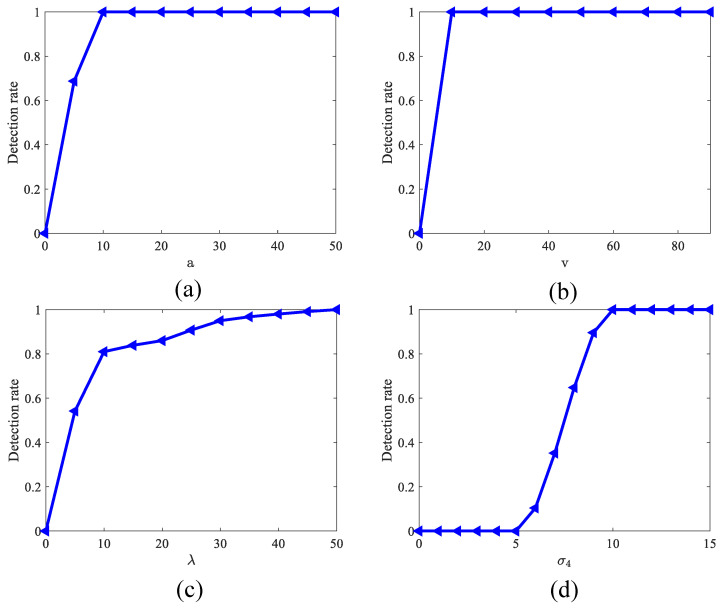
The effects of different model parameters on model performance: (**a**) feedback coefficient *a*; (**b**) range threshold ν; (**c**) inhibition parameter λ; (**d**) spatial scale $\sigma_{4}$. Source: author’s contribution.

$F_{\mathrm{suppressed}}\left(x,y,t\right)$ corresponds to the system’s final processed output. The parameter λ controls the inhibition strength applied to background responses. When the inhibition strength is too weak, false positive responses induced by complex backgrounds cannot be sufficiently suppressed; conversely, excessively strong inhibition may attenuate true small-target responses, thereby degrading detection performance. B(x,y) represents the estimated background component, and $G_{\sigma_{4}}$ denotes a Gaussian kernel with standard deviation $\sigma_{4}$, which controls the spatial smoothing scale. A larger $\sigma_{4}$ corresponds to a broader spatial receptive field and is suitable for modeling slowly varying large-scale background information, whereas a smaller $\sigma_{4}$ preserves more local details. By appropriately selecting $\sigma_{4}$, the model can effectively distinguish spatially diffuse background responses from spatially localized target responses, thereby suppressing background noise without excessively attenuating true small-target signals. This global inhibition mechanism significantly reduces responses in non-target regions while preserving salient features of small targets, thereby enhancing the sensitivity and accuracy of the model in detecting relevant targets. The values of the inhibition strength parameter λ and the spatial smoothing scale parameter $\sigma_{4}$ are determined through parametric sensitivity experiments, as detailed in Figure 6 of the parameters setting section. To detect small objects, we monitor the $F_{\mathrm{suppressed}}\left(x,y,t\right)$ at every time step *t* and compare it to a threshold υ. A small object is flagged when the output at pixel $\left(x_{0},y_{0}\right)$ at time $t_{0}$ exceeds the threshold.

### 2.2. Experimental Dataset

To comprehensively evaluate the effectiveness and generalization ability of the proposed RFSTMD model across diverse scenarios, extensive experiments were performed using two categories of datasets: a self-generated synthetic dataset and video datasets captured in natural environments. The synthetic dataset was specifically created for this study using the Vision Egg software [45]. It consists of video sequences with a frame rate of 1000 Hz, each containing 900 frames. Target sizes vary from 1×1 to 11×11 pixels, and the dataset includes a range of background conditions to thoroughly assess the feasibility and stability of the proposed model.

For real-world validation, we utilized the publicly available RIST dataset [46], which contains small targets moving in natural scenes. From this dataset, 750 frames were selected to evaluate model robustness under challenging conditions, including occlusions, camera motion, and variations in illumination. In these sequences, target sizes range from 3×3 to 15×15 pixels. For transparency and reproducibility, the official RIST dataset is accessible at https://sites.google.com/view/hongxinwang-personalsite/download (accessed on 6 April 2020).

In addition, our self-generated synthetic dataset, along with the full MATLAB source code and detailed implementation instructions, has been publicly released on our GitHub repository: https://github.com/Alingjun920920/RFSTMD-Code-data (accessed on 10 February 2026). All experiments were conducted in MATLAB R2021b on a high-performance computer equipped with a 3.10 GHz Intel i7 CPU and 16 GB of RAM, running Windows 10.

### 2.3. Parameter Setting

Table 2 provides a comprehensive listing of the RFSTMD parameters. The parameters of the motion detection module in our model are categorized into two primary types. The first category comprises target-independent parameters, including $\sigma_{1},n_{1},\tau_{1},n_{2},\tau_{2}$. These parameters are typically preset based on system factors such as sampling rate and video resolution; they define the model’s fundamental operational behavior without relying on specific target attributes. They directly govern the processing of input data, thereby significantly influencing both detection accuracy and computational efficiency. Proper configuration of these parameters enhances the model’s robustness and adaptability, whereas inappropriate settings may compromise detection performance. The second category includes target-prior-dependent parameters, such as $n_{3},\tau_{3},\varphi ,\psi ,e,\rho ,\sigma_{2},\sigma_{3}$. These must be determined in advance based on prior knowledge of the moving targets. Within the model, they control the selective response to target size and velocity. Specifically, $\varphi ,\psi ,e,\rho ,\sigma_{2},\sigma_{3}$ regulate size selectivity, while $n_{3}$ and $\tau_{3}$ primarily determine velocity selectivity. To ensure optimal model output, these parameters must be carefully matched to the actual size and speed of the targets. Furthermore, parameters such as Gaussian kernel sizes, temporal window lengths, and integration constants are critical for defining the spatiotemporal scales of information processing, ensuring the model accurately captures motion features while effectively suppressing background noise. Through empirical tuning, we have refined these parameters to maintain high detection sensitivity and robust performance across diverse scenarios.

Feedback attention pathway and global inhibition modules involve the feedback coefficient *a*, range threshold *v*, inhibition parameter λ, and smoothing scale $\sigma_{4}$. These four parameters were selected based on a parametric sensitivity analysis. To evaluate their impact on model performance, we conducted experiments by systematically varying each parameter and using the detection rate as the performance metric. The results are illustrated in Figure 6a–d. As illustrated in Figure 6a, the model performance effectively reaches saturation when the feedback coefficient hits 10; beyond this value, the performance remains stable. Considering the variations in signal intensity across different video scenarios, we set the feedback coefficient to 30 for subsequent experiments to ensure the feedback mechanism provides sufficient gain even in complex backgrounds. This choice maintains peak performance while further enhancing the model’s robustness against input fluctuations. Similarly, as shown in Figure 6b, the model performance peaks at a range threshold of 10 and then enters a plateau. Although saturation is achieved at 10, we fixed the range threshold at 35 for all following experiments to maintain a larger tolerance margin when handling various types of complex noise. Experimental results demonstrate that this configuration ensures optimal and stable detection performance across diverse test conditions. Figure 6c illustrates the network’s sensitivity to the global inhibition parameter λ. It is observed that as the inhibition parameter increases from 0 to 50, the detection performance improves progressively, reaching its peak at 50. This result indicates that setting the inhibition parameter to 50 most effectively suppresses background false positives. Consequently, we fixed this parameter at 50 in our subsequent experiments. Figure 6d illustrates the network’s sensitivity to the spatial scale parameter $\sigma_{4}$ used for background estimation. It can be observed that when the spatial scale $\sigma_{4}$ ranges from 0 to 5, the model’s detection performance is zero. This is because the model is tuned for a preferred small-target size of 5×5 pixels; at smaller spatial scales, the Gaussian kernel fails to effectively estimate the background as it tends to suppress the target itself. As the spatial scale increases, the detection rate improves progressively, reaching a peak at $\sigma_{4}=10$. Beyond this point, detection performance remains relatively stable. This peak indicates that setting $\sigma_{4}$ to 10 or slightly above allows the model to effectively isolate background false positives while preserving target integrity. Therefore, in all subsequent experiments, we set the spatial scale parameter to 15 (slightly above 10) to maintain optimal and robust detection performance.

### 2.4. Experimental Setup

**Figure 7:**
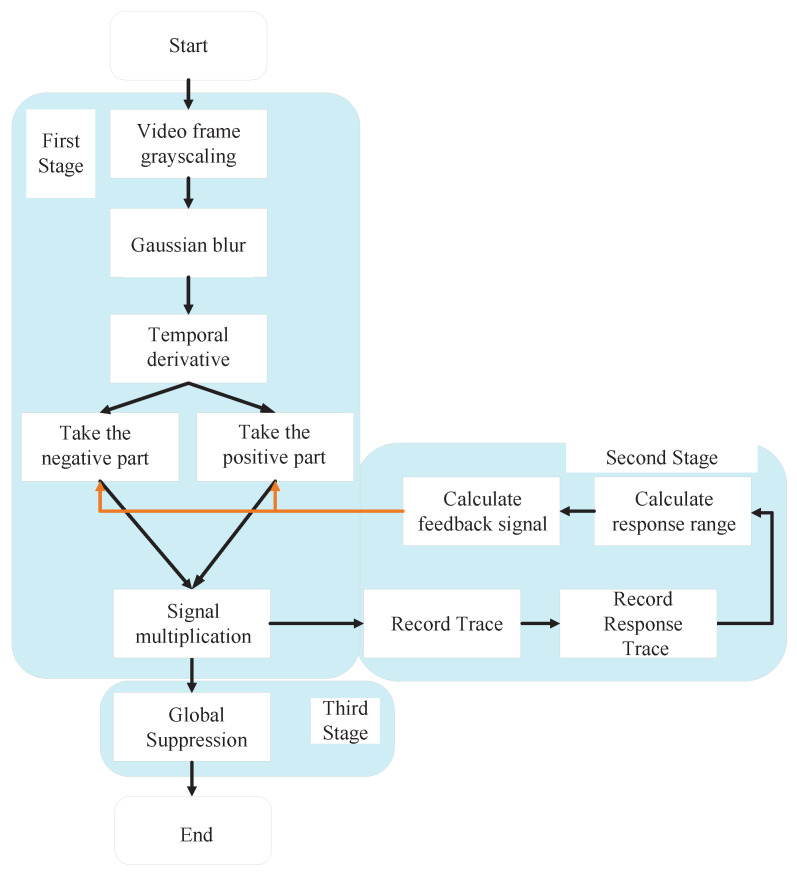
Flowchart of the model implementation algorithm. Black arrows indicate the information transmission process, while red arrows indicate the feedback process. Source: author’s contribution.

The implementation of the RFSTMD model is organized into a three-stage computational pipeline, as illustrated in Figure 7.

Stage 1: Motion Feature Extraction and Preprocessing

**Figure 8:**
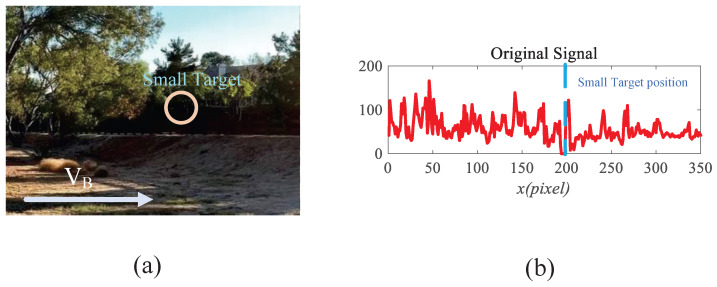
(**a**) A dataset frame at t=580 ms depicts a small target traversing in the opposite direction to a complex dynamic background, where the arrow indicates the motion direction of the background. (**b**) The input information. The red line represents input response, and vertical dashed line represents the position of small target. Source: author’s contribution.

**Figure 9:**
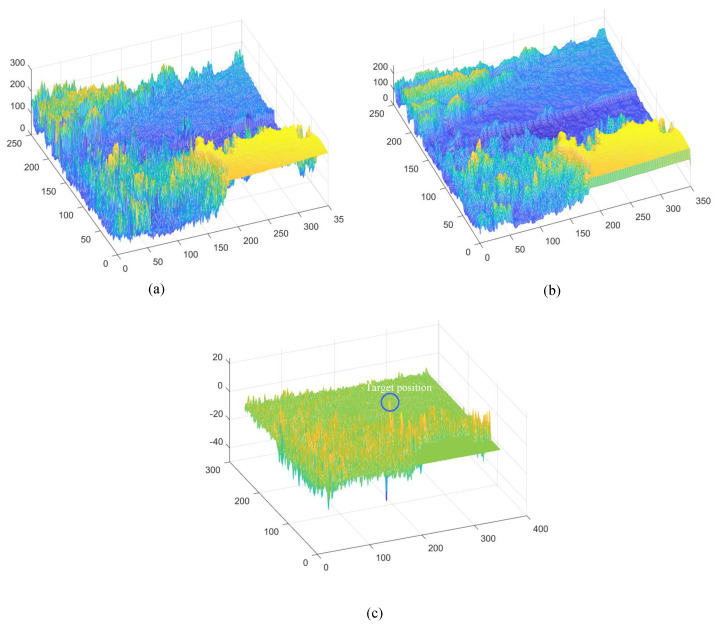
(**a**) Grayscale image matrix. (**b**) Ommatidia response matrix. (**c**) LMC response matrix, where the blue circle indicates the positive response output at the small target positions, and the blue line below indicates the negative response output at the same positions. Source: author’s contribution.

This stage transforms raw video inputs into neural representations (see Figure 8). The input video sequence is first decomposed into consecutive frames, each of which is converted to grayscale to serve as the primary luminance signal (see Figure 9a). According to Equation (1), the frames are convolved with a Gaussian kernel for spatial smoothing, thereby suppressing sensor noise while preserving target structural information (see Figure 9b).

**Figure 10:**
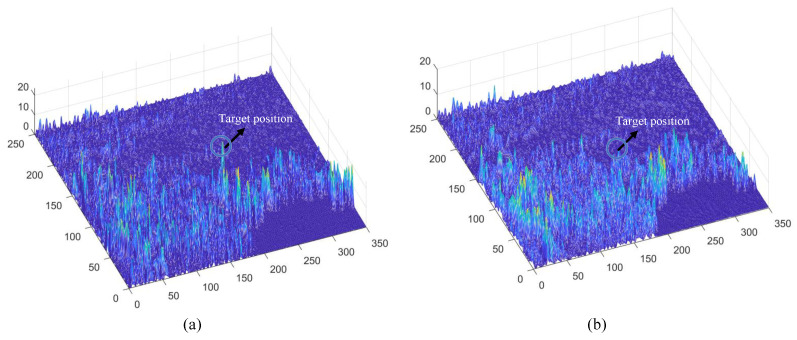
(**a**) Tm3 response matrix, where the circle represents the small target position. (**b**) Tm1 response matrix, where the circle represents response output of the small target position. Source: author’s contribution.

**Figure 11:**
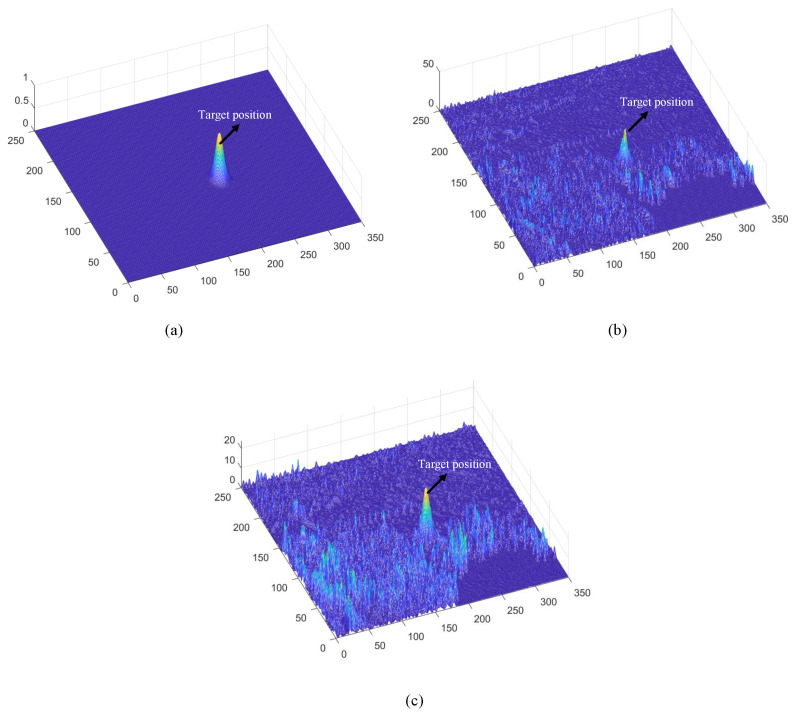
(**a**) Feedback signal, where the colored highlighted areas indicate the positions of the small targets. (**b**) Feedback Tm3 response, where the colored highlighted areas indicate the positions of the small targets. (**c**) Feedback Tm1 response, where the colored highlighted areas indicate the positions of the small targets. Source: author’s contribution.

Subsequently, Large Monopolar Cells (LMCs) extract temporal luminance variation using Equation (3) (see Figure 9c), and the resulting signal is separated into positive and negative components (see Figure 10a,b). A temporal delay is applied to the negative channel, while the feedback signal from the previous frame (initialized to zero, see Figure 11a is integrated into both channels (see Figure 11b,c). Finally, STMD neurons perform multiplicative integration followed by convolution with an inhibitory kernel to generate the initial motion detection response.

Stage 2: Feedback Attention Pathway

**Figure 12:**
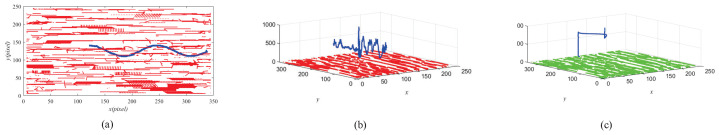
(**a**) Motion trace (blue line represents true target, red lines represent false positives). (**b**) Response trace (blue line represents true target, red lines represent false positives). (**c**) Response range (blue line represents true target, red lines represent false positives). Source: author’s contribution.

This stage implements the central innovation of the proposed method (Figure 12), using spatiotemporal differential cues to distinguish true targets from background clutter. The workflow consists of three sequential steps:

Trajectory Estimation: Motion trajectories are estimated via neighborhood matching between consecutive frames. If a detection point in frame t+1 falls within the predefined spatial neighborhood of a point in frame *t*, the two points are associated with the same trajectory.

Response Analysis: For each trajectory, temporal response sequences are extracted across frames to compute the temporal response range.

Threshold Logic: The variation range is compared with a predefined threshold to identify true target trajectories. Feedback signals are subsequently generated at these locations to provide high-precision guidance for detection in the subsequent frame.

Stage 3: Global Suppression and Validation

**Figure 13:**
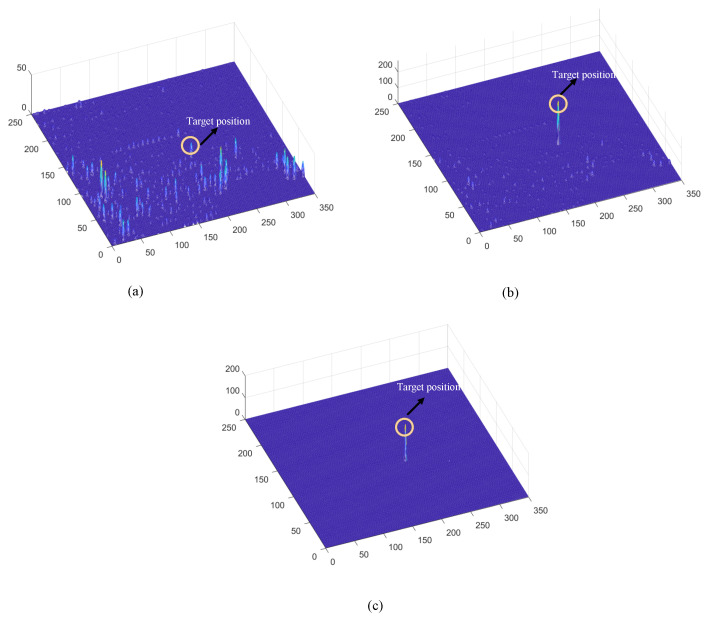
(**a**) ESTMD response, where the lines inside the orange circles indicate the responses at the positions of the small targets. (**b**) Without global suppression response, where the line inside the orange circles indicates the responses at the positions of the small targets. (**c**) With global suppression response, where the line inside the circles indicates the responses at the positions of the small targets. Source: author’s contribution.

This stage further improves detection accuracy by eliminating background interference. The background feature response is estimated from the detection output and subtracted from the current detection map, thereby reducing background noise (Figure 13c). The three-stage pipeline ensures that the model maintains high robustness and detection accuracy under complex and dynamic background conditions.

Based on the outputs of each stage, the model effectively validates the two initial hypotheses: first, the feedback attention pathway can reliably distinguish small targets from background clutter, enhancing responses to true targets; second, the coordinated interaction between the feedback pathway and the global inhibitory mechanism significantly improves detection accuracy, demonstrating the effectiveness and consistency of the proposed framework in tiny object detection.

## 3. Results

This section validates the effectiveness of the proposed model through experimental evaluation and presents comparative performance results to demonstrate its advantages.

### 3.1. Performance Evaluation of RFSTMD

To gain deeper insight into the operational dynamics of RFSTMD, synthetic sequences produced by Vision Agg were employed to visualize its information-processing procedure. Figure 8a depicts a representative frame where a small target navigates a complex background at speeds of 250 and 150 pixels per second. The background motion, shown by vector $V_{B}$, is directed oppositely to the target’s movement. To verify the implementation of the mathematical formulations, the intermediate outputs of the current frame are presented to provide an intuitive illustration of the processing results (see Figure 9, Figure 10, Figure 11, Figure 12 and Figure 13). Figure 9a,b show the grayscale input frame and the corresponding ommatidia response. The ommatidia output is spatially smoothed using a Gaussian filter to suppress high-frequency noise. Figure 9c presents the output of the LMC, obtained by extracting luminance changes. The output comprises both positive and negative values, where positive values represent increases in luminance and negative values represent decreases. Figure 10a,b presents the response outputs of the Tm3 and Tm1 neurons. Tm3 is sensitive only to luminance increments, and its response is derived from the positive component of the LMC output. Tm1, on the other hand, is sensitive to luminance decrements, and its response is obtained from the negative component of the LMC output after applying a temporal delay.

Before the STMD neurons integrate the outputs of Tm3 and Tm1, Tm3 and Tm1 first pre-integrate the feedback signal from the previous time step. The generation of this feedback signal follows the following logic: first, background false positives are removed and small targets are identified based on response time differences from the previous outputs; then, feedback is generated at the corresponding locations of the small targets to enhance features in the subsequent time step (see Figure 12). Figure 12a shows the recorded motion trajectories. It can be seen that, due to the high complexity of the trajectory information, relying solely on spatial motion features is insufficient to effectively eliminate background false positives. Figure 12b presents the temporal response curves computed based on the motion trajectories. The results indicate that the responses of small targets exhibit pronounced temporal fluctuations, whereas the responses of background false positives remain relatively stable. Accordingly, the temporal response range can serve as a key feature to distinguish between the two. Figure 12c shows the distribution of the computed response range, demonstrating that by setting an appropriate range threshold, small targets can be accurately identified while suppressing background noise, thereby providing a reliable basis for subsequent motion target detection. After using the response range to distinguish small targets from background false positives, the system generates a feedback signal matrix at the small-target locations, formed by a Gaussian function (see Figure 11a). It can be observed that the feedback signal appears only at the small-target locations, indicating that using the response range to differentiate small targets from background false positives is effective. Furthermore, the Tm3 and Tm1 neurons integrate the feedback signal to form new response outputs (see Figure 11b,c). The results show that the newly generated Tm3 and Tm1 responses are significantly enhanced at the small-target locations, while the responses at other locations remain unchanged. Figure 13a shows the initial response output. It can be seen that in the original output, the responses of small targets are relatively weak and are accompanied by substantial background false-positive interference. Figure 13b presents the responses after introducing the feedback mechanism, where the responses at the small-target locations are significantly enhanced, although some false-positive responses still remain. Figure 13c illustrates the final responses of the complete model. After global inhibition, background false positives are effectively suppressed, leaving only clear small-target responses, whose amplitudes are considerably higher than those in the original output. These results demonstrate that the model can effectively suppress background noise while significantly enhancing selective attention to small targets.

**Figure 14:**
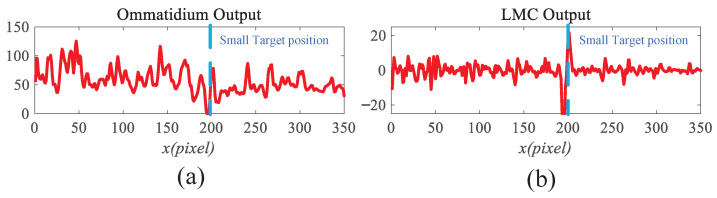
(**a**) The ommatidia response of ESTMD model and RFSTMD model, where red line represent response output and vertical dashed line represents the response of true small target position. (**b**) The LMC response of ESTMD model and RFSTMD model, where red line represent response output and vertical dashed line represents the response of true small target position. Source: author’s contribution.

**Figure 15:**
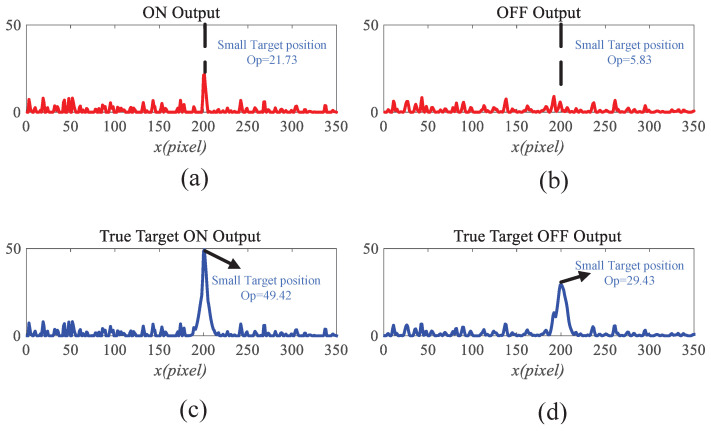
(**a**) The Tm3 response of ESTMD model, where red line represents response output and black dashed line represents the response of true small target position. (**b**) The Tm1 response of ESTMD model, where red line represents response output and black dashed line represents the response of true small target position. (**c**) The Tm3 response of RFSTMD model, where blue line represent response output. (**d**) The Tm1 response of RFSTMD model, where blue line represent response output. Source: author’s contribution.

For clearer visualization of the small-target position response, the time was fixed at $t_{0}=580$ ms, and neural activity was extracted along the vertical position $y_{0}$. Figure 8b displays the captured luminance signal $I(x,y_{0},t_{0})$ together with the corresponding ommatidium output. The input signal is strongly affected by background noise, which makes distinguishing the true target from interference challenging. Figure 14a shows the corresponding ommatidium response $P(x,y_{0},t_{0})$. The ommatidium neurons conduct Gaussian smoothing, which generates a cleaner and smoother output than the input signal. As illustrated in Figure 14b, the LMCs exhibit complex response dynamics arising from temporal variations in ommatidial outputs. Positive values correspond to luminance increases at time $t_{0}$, whereas negative values indicate decreases. Because these plots capture only transient signal fluctuations, they do not highlight features specific to the target, rendering them insufficient for reliable motion detection or false-alarm reduction. Figure 15a,b illustrate the response dynamics of Tm3 and Tm1 neurons in the non-feedback model. These responses are obtained by decomposing the LMC outputs into their positive and negative components. Specifically, the Tm3 neuron output reflects the positive component of the LMC response, predominantly representing increases in luminance. In contrast, the Tm1 neuron encodes the delayed negative component, corresponding to decreases in luminance. As illustrated in Figure 15c,d, Tm3 and Tm1 neuron responses are observed under the feedback-enhanced configuration. It can be observed that at the true small-target location, the responses of both Tm3 and Tm1 neurons are significantly strengthened due to the introduction of feedback signals, while almost no changes occur at non-target regions.

**Figure 16:**
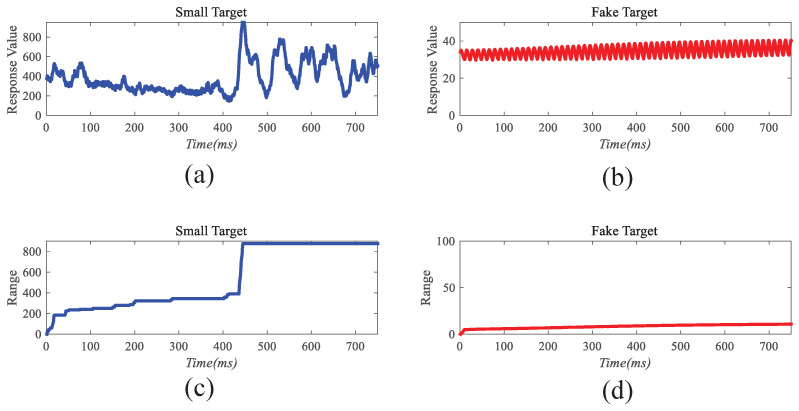
(**a**) The temporal response of the small target. (**b**) The temporal response of the fake target. (**c**) The range of small target with time *t*. (**d**) The range of fake target with time *t*. Source: author’s contribution.

**Figure 17:**
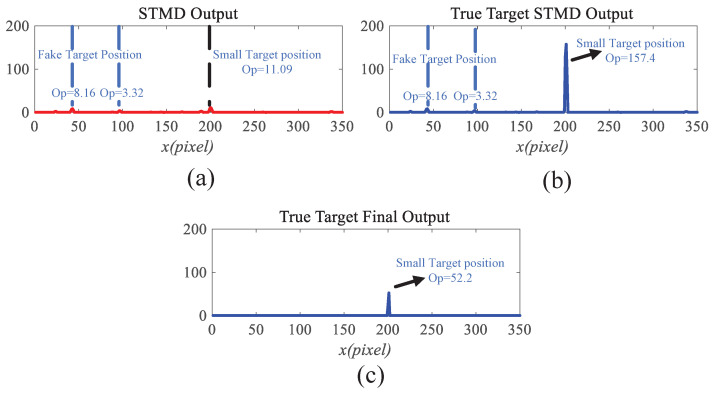
(**a**) ESTMD response. The red line represents response output, black dashed line represents the response of true small target position, and blue dashed lines represent the response of fake target position. (**b**) RFSTMD response, where blue line represents response output, blue dashed lines represent the response of fake target position. (**c**) RFSTMD final response, where blue line represent response output. Source: author’s contribution.

To clearly illustrate the information transmission process within the feedback pathway, we selected the response at a real small-target location and that at a background false-positive location for comparison. Figure 16a,b show the temporal variations of the responses corresponding to the real small target and the background false positive, respectively. It is evident that the small target’s response exhibits pronounced temporal fluctuations, while the response of false positives remains comparatively stable. This contrast enables effective discrimination of small targets from false positives. Furthermore, Figure 16c,d depict the temporal variations of the response ranges for both cases. It is observed that the small target’s response range grows gradually over time, in contrast to the nearly stable response range of the false positive. By exploiting this discriminative characteristic, the small-target position can be accurately identified, and feedback information is generated at that location using a Gaussian function to enhance the model’s attention to the small target. In contrast, no feedback is applied at the background false-positive location, so its final response remains unchanged. Figure 17a,b shows the fusion results of STMD outputs derived from Tm3 and Tm1 neurons in both the feedback and non-feedback models. A comparison reveals that the feedback mechanism effectively enhances the responses of Tm3 and Tm1 neurons at target positions, thereby improving the model’s selectivity and attentional focus on small targets, and ultimately enhancing detection saliency and robustness. A comparison of the results with and without feedback reveals that, although the feedback mechanism enhances attention to small targets, false-positive features in the background still persist. To effectively suppress these background interferences, the feedback model further implements a large-scale inhibition mechanism. Figure 17c illustrates the outputs of the feedback model after applying this global suppression. Observations indicate that responses from background false positives are greatly diminished, while responses at true small-target positions are preserved. These experimental results demonstrate that our model not only enhances attention to small targets but also effectively distinguishes real targets from complex background noise, thereby further improving detection accuracy and robustness.

### 3.2. The Effectiveness of Global Suppression

**Figure 18:**
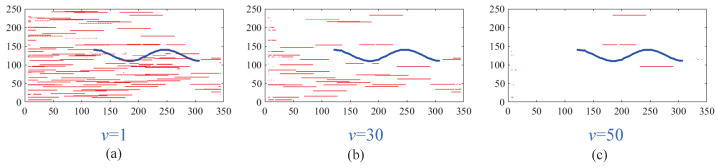
Recorded target trajectories of our model under different global suppression parameters, where the blue line represents the motion trajectories of small targets, and the red lines represent false positive trajectories. (**a**) ν=1; (**b**) ν=30; (**c**) ν=50. Source: author’s contribution.

**Figure 19:**
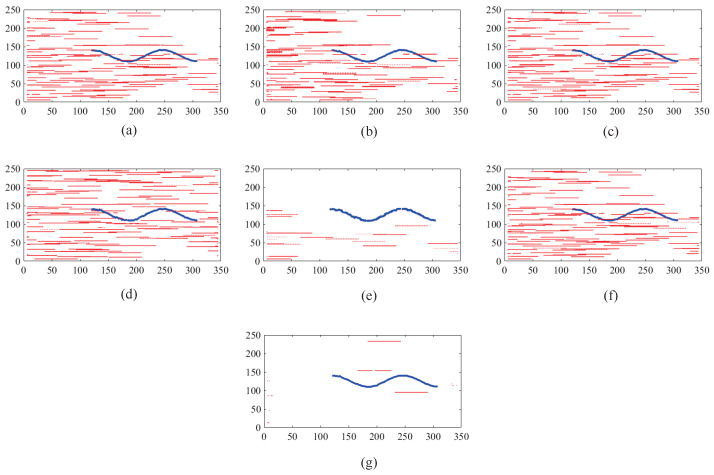
Recorded motion trajectories of all benchmark models, where the blue line represents the motion trajectories of small targets, and the red lines represent false positive trajectories. (**a**) ESTMD; (**b**) DSTMD; (**c**) FSTMD; (**d**) Feedback STMD; (**e**) Frac-STMD; (**f**) ST-STMD; (**g**) RFSTMD. Source: author’s contribution.

**Figure 20:**
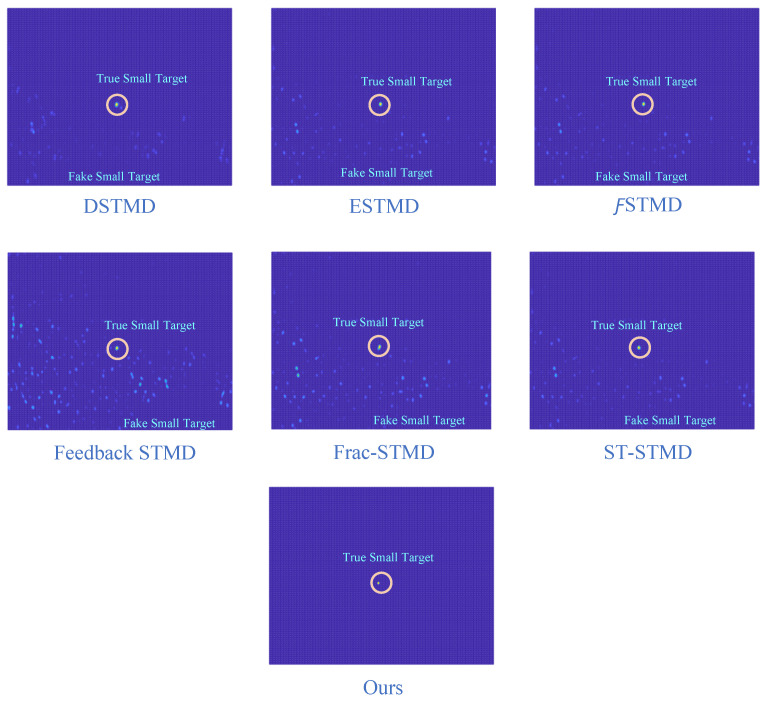
2D visualizations of various detection methods. Source: author’s contribution.

In this study, we introduced a global suppression mechanism at the final stage of the STMD neural network output to mitigate background false-positive responses. To assess its effectiveness, we evaluated different suppression parameters and examined the resulting feature trajectories. Motion trajectories of small targets (blue) and false positives (red) at different ν thresholds are illustrated in Figure 18. At lower threshold values, many false trajectories persist; however, as the threshold rises, their occurrence gradually diminishes, highlighting the critical role of the threshold in regulating the false alarm rate. With higher threshold settings, true target trajectories can be more reliably distinguished, resulting in a lower incidence of false alarms. The results demonstrate that the proposed network efficiently reduces background false positives, markedly enhancing both the accuracy and robustness of small-target detection. Figure 19 presents the motion trajectories obtained from several comparative models in addition to the proposed RFSTMD. Trajectories produced by the comparative models contain numerous spurious features, complicating reliable target identification. By employing a global suppression threshold, the proposed model effectively mitigates background false positives. Consequently, the detected trajectories closely align with the actual paths of the small targets, with only minimal extraneous responses remaining. This approach substantially decreases false detections while simultaneously improving detection precision and stability. Furthermore, Figure 20 shows the final detection results for several benchmark models. It is evident that the outputs of alternative models include a significant number of false positives, thereby limiting their ability to clearly distinguish targets from the background. By effectively attenuating background interference, the proposed model retains only the responses of genuine small targets. These observations substantiate that our approach ensures reliable and precise detection of tiny targets even amidst complex backgrounds.

### 3.3. Ablation Study

**Figure 21:**
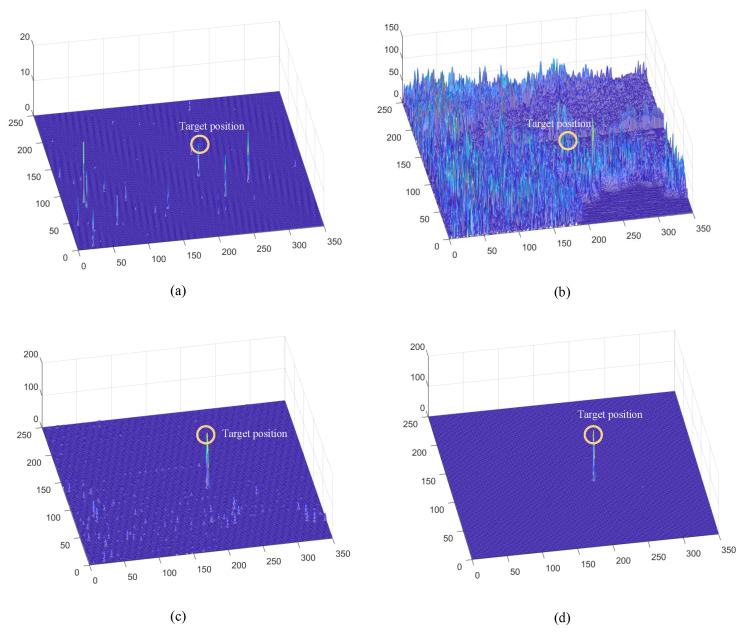
(**a**) Without feedback output. (**b**) Without motion detection pathway output. (**c**) Without global suppression pathway output. (**d**) RFSTMD output. The circles indicate the responses at the small target positions. Source: author’s contribution.

Ablation experiments were conducted to evaluate the contribution of each module to the overall system performance, with qualitative results shown in Figure 21. When the feedback pathway is removed, the system’s gain modulation is significantly reduced, resulting in extremely weak responses at target locations (maximum value below 20) and numerous false-positive signals in the background, highlighting the critical role of feedback in enhancing weak target signals (Figure 21a). Removing the motion detection module causes the model to lose its ability to extract spatiotemporal motion features and suppress static large targets, leading to cluttered outputs that retain most of the original interference and fail to separate targets from the background (Figure 21b). In the absence of the global suppression mechanism, feedback can moderately enhance target responses; however, due to the lack of global inhibition, substantial false-positive responses still remain in the background (Figure 21c). Finally, when all modules are activated, the collaborative operation of motion detection, feedback enhancement, and large-scale suppression enables optimal detection performance (Figure 21d), effectively suppressing background interference while preserving high signal-to-noise responses only at small-target locations, thereby validating the indispensable contribution of each module to the overall robustness and effectiveness of the system.

### 3.4. Performance Comparison

In this subsection, we investigate the performance of the proposed RF-STMD network by conducting an extensive comparative study. Its ability to detect small moving targets is quantitatively evaluated by comparing it with six representative STMD-based methods, namely ESTMD, DSTMD, F-STMD, Feedback STMD, Frac-STMD, and ST-STMD. All models are assessed under the same experimental settings, enabling a fair and systematic comparison between the proposed approach and existing state-of-the-art techniques. Model performance is quantitatively analyzed using the Receiver Operating Characteristic (ROC) curve, where detection accuracy $D_{A}$ is examined as a function of the false alarm rate $F_{A}$. Specifically, $D_{A}$ quantifies the proportion of small targets that are successfully detected, while $F_{A}$ measures the rate at which non-target background elements are erroneously classified as targets. Employing ROC curves provides a comprehensive view of the trade-off between sensitivity and specificity, thereby enabling a clear and quantitative comparison of the different STMD-based models. Specifically, $D_{A}$ and $F_{A}$ are defined as follows:

(20) $$D_{A}=\frac{true\;detections}{total\;targets},\;\;F_{A}=\frac{false\;detections}{image\;count}.$$

In this notation, true detections is the number of correctly identified small targets, total targets is the total ground-truth count, false detections is the number of false positives, and image count is the total number of frames.

The network’s performance was initially tested across simulations with different target velocities, sizes, luminance levels, and background motion patterns, and benchmarked against several established frameworks. Details of the simulated frame sequences are listed in Table 3.

**Figure 22:**
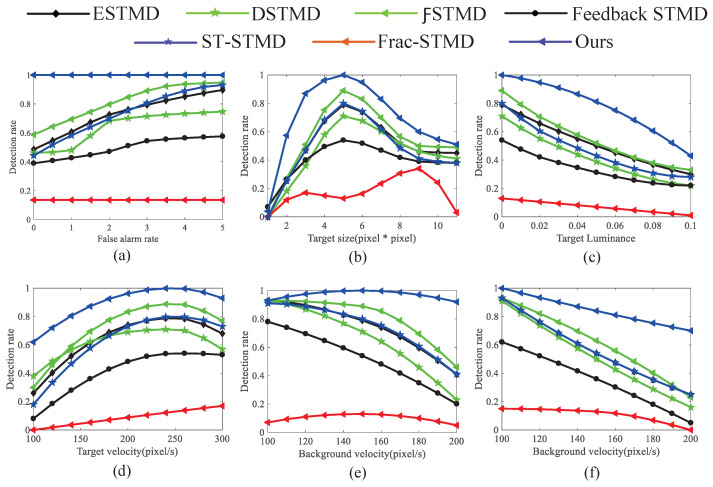
(**a**) ROC curves showing small-target detection for seven benchmark models on the original test set. (**b**–**f**) ROC curves of the seven models across datasets varying in target size, brightness, speed, and background motion. Source: author’s contribution.

Figure 22a displays ROC curves comparing the detection capabilities of various models on simulated sequences. The RF-STMD model demonstrates consistently higher detection performance across a range of false-alarm levels, indicating its superiority over existing approaches. This result confirms that incorporating feedback and large-scale suppression mechanisms effectively mitigates background false positives, leading to a substantial enhancement in detection precision. The ROC curves in Figure 22b–f illustrate the performance of various benchmark networks across different simulation conditions, holding the false alarm rate $F_{A}=3$. Specifically, Figure 22b presents the detection performance for targets of different sizes. Most models perform best when the target size lies between 3×3 and 8×8 pixels. By contrast, the proposed model, benefiting from the integration of feedback and large-scale suppression mechanisms, exhibits markedly improved detection capability over the six alternatives. These findings indicate that the proposed framework substantially enhances the discrimination of tiny targets and maintains superior detection accuracy across diverse target sizes, thereby validating its effectiveness in complex background scenarios. Figure 22c compares the detection results of several models under varying target brightness conditions. Across a luminance range of 0 to 0.1, all seven models experience a reduction in ROC values, but the RF-STMD model consistently demonstrates the highest detection accuracy. Overall, these results demonstrate the high robustness of our model in detecting small targets within complex environments, and they highlight the essential contribution of feedback and large-scale suppression mechanisms to enhancing both detection accuracy and system stability. Figure 22d shows the detection performance of various models across different target velocities. Across the 0–300 pixels/s velocity range, the RF-STMD model consistently achieves superior ROC performance compared with other models, attaining higher detection accuracy at the same velocity. These results indicate that the combined use of feedback and large-scale suppression enhances motion feature extraction, suppresses background dynamics, and boosts robustness and accuracy across various velocities. Figure 22e,f provide detection results under diverse background velocities and motion directions. Across different background dynamics and object trajectories, the RFSTMD model consistently outperforms other methods in detection. These results emphasize that integrating feedback with large-scale suppression helps the model differentiate target motion from background, improving detection reliability in complex environments.

**Figure 23:**
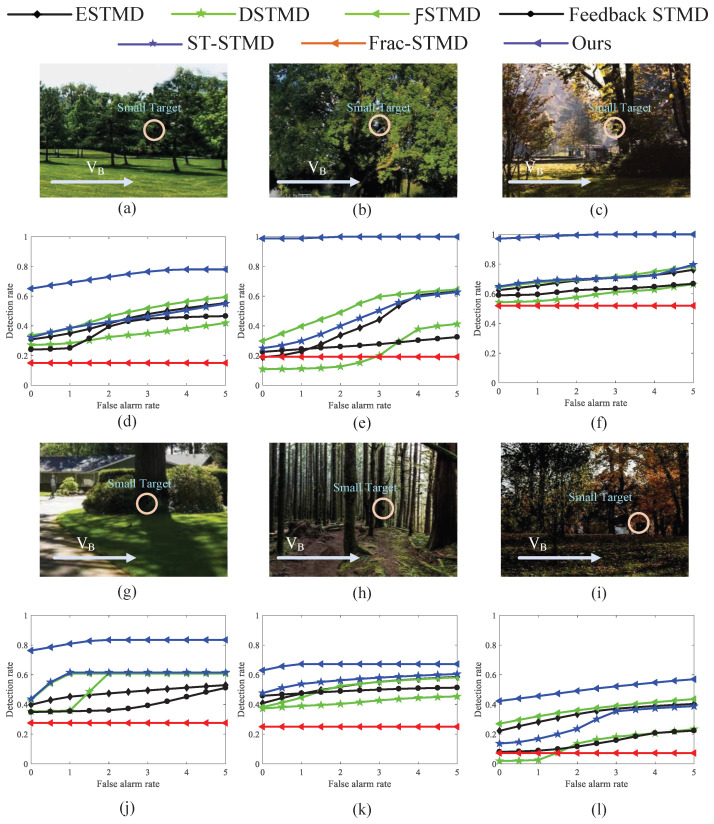
(**a**–**c**,**g**–**i**) Example frames from six simulated datasets. (**d**–**f**,**j**–**l**) ROC curves comparing benchmark models with the RFSTMD method for small-target detection. Source: author’s contribution.

**Figure 24:**
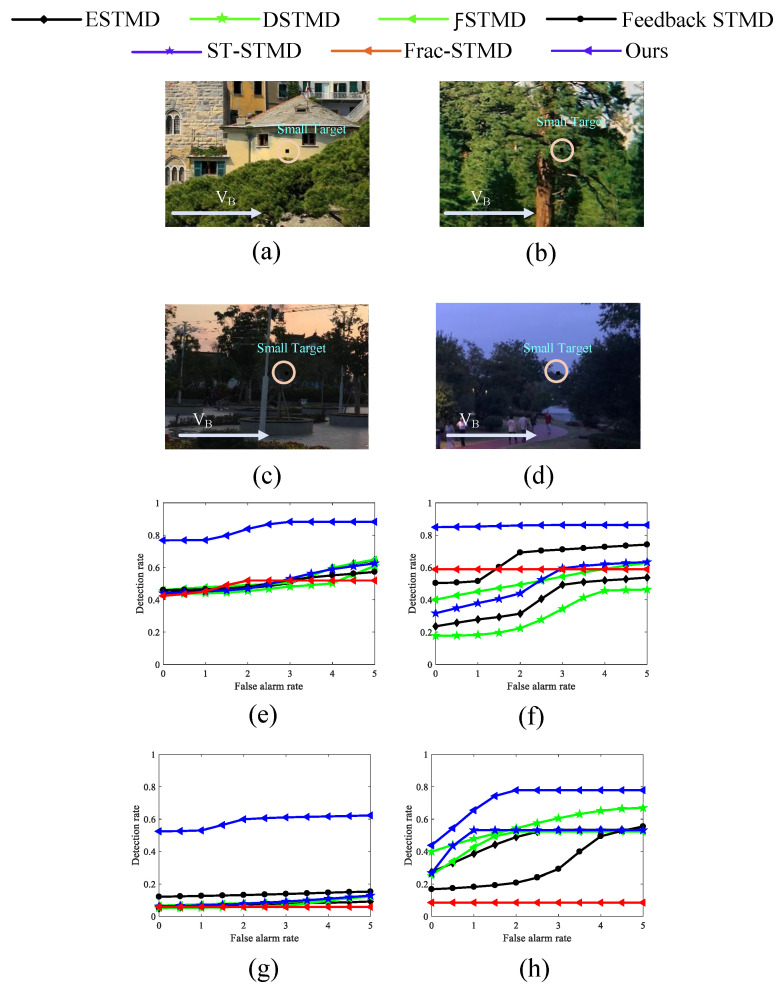
(**a**,**b**) Example frames from high contrast simulated datasets. (**c**,**d**) Example frames from low contrast simulated datasets. (**e**–**h**) ROC curves comparing benchmark models with high contrast and low contrast simulated datasets. Source: author’s contribution.

**Figure 25:**
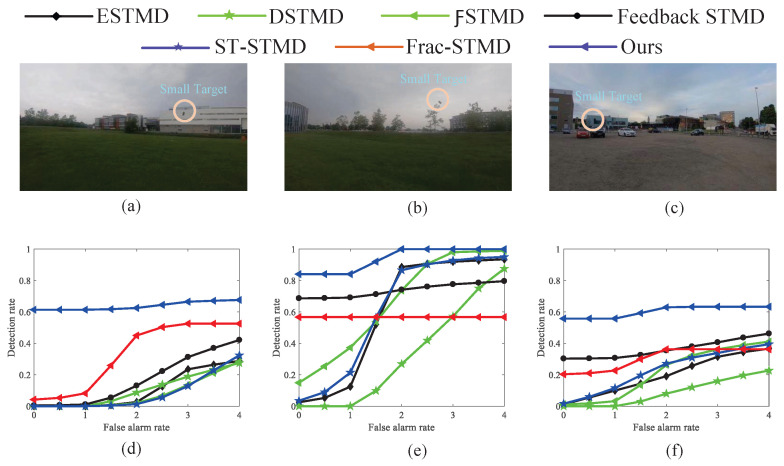
(**a**–**c**) Example frames from three real-world datasets. (**d**–**f**) ROC curves comparing benchmark models with RFSTMD for small-target detection. Source: author’s contribution.

Figure 23 provides ROC curves for benchmark models assessing real small-target detection under complex motion conditions, highlighting comparative performance. Figure 23a–c,g–i provide example frames of small-target motion, with the background direction indicated by $V_{B}$ and the target moving oppositely. Figure 23d–f,j–l summarize the detection performance of seven models under dynamic, complex backgrounds. These results provide clear evidence that the proposed visual neural network outperforms six benchmark models across all test conditions, delivering higher detection performance and reduced false positives, highlighting its reliability and robustness in complex small-target detection tasks. Figure 24 presents the ROC curves of various benchmark models for real small-target detection under complex motion conditions with high contrast and low illumination, providing a comparative evaluation of different methods. The experimental results indicate that RFSTMD is able to maintain stable detection performance under these challenging conditions and exhibits clear performance advantages over existing STMD models. Additionally, Figure 25 illustrates the RF-STMD model’s performance on real-world datasets, including sample frames and a comparison across seven reference models. Figure 25a–c show sample frames from three different real-world datasets, while Figure 25d–f present ROC curves for the benchmark models under various practical conditions. The results demonstrate that the RF-STMD model consistently achieves higher detection accuracy than other networks.

Table 4 presents a quantitative comparison of the detection performance. The results demonstrate that at a false alarm rate of 3, our model achieves an average detection rate of 0.81 (95% confidence interval: 0.81 ± 0.131), which significantly outperforms all competing methods—including DSTMD (0.45 ± 0.216), ESTMD (0.53 ± 0.155), FSTMD (0.58 ± 0.179), Feedback STMD (0.45 ± 0.128), Frac-STMD (0.31 ± 0.130), and ST-STMD (0.54 ± 0.170)—all of whose detection rates remain below 0.59. This substantial margin underscores the superior robustness of our model in detecting small moving targets under challenging conditions. Finally, the models are evaluated in terms of precision and F1-score, with the corresponding results summarized in Table 5 and Table 6, providing a comprehensive comparison. It is evident that our model achieves superior suppression of background false alarms, attaining an average precision of 0.0648 (95% confidence interval: 0.0648 ± 0.021). This performance markedly surpasses other state-of-the-art models—specifically DSTMD (0.0077 ± 0.002), Frac-STMD (0.0074 ± 0.002), ST-STMD (0.0062 ± 0.001), ESTMD (0.0061 ± 0.001), FSTMD (0.0061 ± 0.001), and Feedback STMD (0.0058 ± 0.001)—all of whose average precision remains below 0.008. Furthermore, the proposed model outperforms alternative methods in terms of F1-score, achieving an average of 0.12 (95% confidence interval: 0.12 ± 0.038), whereas other methods remain below 0.02—specifically DSTMD (0.015 ± 0.003), Frac-STMD (0.015 ± 0.004), ESTMD (0.013 ± 0.002), F-STMD (0.012 ± 0.002), ST-STMD (0.012 ± 0.002), and Feedback ESTMD (0.011 ± 0.002). This significant improvement reflects enhanced moving target identification and a superior trade-off between precision and recall. The experimental results indicate that the RFSTMD model achieves enhanced robustness and stability across a wide range of dynamic background scenarios and different target types, with performance consistency further supported by the reported confidence intervals.

## 4. Discussion

This section discusses the experimental results and analyzes the limitations of the model as well as potential directions for future research.

### 4.1. Operational Mechanism of Feedback Attention

This study employs visualization analysis to provide an in-depth explanation of the operational mechanism of the Feedback Attention Mechanism. The mechanism exploits the intrinsic differences in spatiotemporal response characteristics between small targets and background false positives to achieve selective enhancement of target signals. Specifically, experimental observations show that at true small-target locations, STMD neuron responses exhibit pronounced temporal fluctuations and gradually increase over time, whereas responses at background false-positive locations remain relatively stable (see Figure 16). These results validate Hypothesis 1 proposed in this work, namely that the response differences induced by moving targets and background false positives can serve as key cues for the nervous system to distinguish signals from noise. At the implementation level, when such spatiotemporal differential features are detected, the feedback mechanism injects Gaussian Feedback Gain at the corresponding small-target locations (see Figure 15c,d), thereby significantly enhancing the sensitivity of STMD neurons to small targets. This iterative “detection–guidance–enhancement” strategy effectively emulates the top-down modulation mechanism observed in biological visual systems, enabling the model to maintain sustained and precise attention to targets in complex backgrounds. However, comparative experiments also reveal the limitations of this mechanism: although feedback enhancement alone can substantially boost target responses, it remains insufficient to completely eliminate all residual background activity (see Figure 17b). This finding further underscores the necessity of incorporating a global suppression mechanism into the model architecture to achieve more thorough target–background separation.

### 4.2. Background Suppression via Global Suppression

To address these challenges, this study introduces a global suppression mechanism at the model’s output stage. This mechanism effectively filters out interference from dynamic backgrounds (see Figure 17). Experimental results show that increasing the suppression parameter significantly reduces false responses caused by background clutter. Meanwhile, the gain protection provided by the feedback mechanism ensures that responses to true targets are substantially enhanced and fully preserved, achieving a balance between detection accuracy and false alarm rate. Visualization results further confirm the effectiveness of this design. After global suppression, background false positives are significantly diminished, while responses to true targets remain clear (see Figure 17c), and the final output contains only pure target signals (see Figure 17c and Figure 20). Comparative experiments show that output trajectory maps from other models are often heavily contaminated by false features. In contrast, our model extracts clean and coherent target trajectories that closely match the ground truth paths. This demonstrates that the combined effect of top-down feedback enhancement and global suppression provides significant advantages in suppressing complex background clutter.

### 4.3. Comprehensive Performance Validation

Systematic quantitative comparative experiments further verify the superior performance of RFSTMD. As shown in Figure 22a, at the same false alarm rate levels, RFSTMD consistently achieves the highest detection rate, with its ROC curve significantly outperforming six comparative methods. These results clearly demonstrate that the synergistic integration of feedback attention and global suppression mechanisms effectively reduces background-induced false positives, thereby substantially improving detection accuracy. This finding also validates Hypothesis 2, namely that the proposed model can significantly enhance small-target detection performance.

Even when compared with Feedback STMD, which already incorporates a feedback mechanism, RFSTMD still shows clear advantages. This suggests that temporal feedback modulation alone is insufficient to handle complex background interference. Effective separation between true targets and background clutter requires combining feedback enhancement with global spatial suppression. This insight provides guidance for designing bio-inspired visual systems, highlighting that the synergy between feedback modulation and large-scale suppression may be a key architectural feature for robust target detection.

Further analysis under different simulation conditions reveals that RFSTMD maintains stable performance across variations in target scale, luminance, and motion speed. Regarding target scale (Figure 22b), conventional models usually perform best within the 3×3 to 8×8 pixel range. In contrast, RFSTMD consistently achieves superior performance across the entire tested scale range. This advantage arises from the feedback mechanism’s ability to dynamically enhance weak target responses, effectively overcoming the receptive field scale limitations of traditional STMD models.

Under varying luminance conditions (Figure 22c), although detection performance decreases as luminance drops, RFSTMD consistently maintains the highest accuracy. Here, the feedback mechanism enhances small-target responses through temporal accumulation, while the global suppression mechanism prevents background noise from being misclassified as targets in low-contrast settings.

In terms of speed adaptability (Figure 22d), RFSTMD performs well over a wide velocity range of 0–300 pixels/s. This indicates that the feedback–suppression collaborative architecture can adaptively extract motion differences between targets and backgrounds. Moreover, in challenging scenarios involving varying background speeds (Figure 22e) and diverse motion directions (Figure 22f), the model continues to exhibit stable detection performance, confirming its robustness and adaptability in complex dynamic environments.

### 4.4. Biomimetic Contribution

This study discriminates small targets and enhances attention to them based on the response range. Although the response-range mechanism is implemented as an engineering approximation, its underlying logic closely aligns with the nonlinear processing principles observed in biological visual systems. In biological STMD pathways, target detection does not rely on simple linear accumulation; instead, mechanisms such as shunting inhibition and adaptive gain control enable transient amplification of salient moving targets. In the proposed RFSTMD, the response range, serves as a “computational proxy” for these complex synaptic interactions, emulating the neurons’ “derivative-like” sensitivity to temporal non-stationarity. It provides high gain for targets exhibiting large transient response changes while naturally suppressing steady or slowly varying background signals. This approach embodies the evolutionary principle of energy-efficient computation in neural circuits—enhancing target signals through minimal comparative logic—effectively bridging the gap between strict biomimetic replication and practical computational efficiency in machine vision.

### 4.5. Limitations and Future Directions

The results of this study are highly consistent with the hierarchical processing principles of biological visual systems. Neurons in the primary visual cortex (V1) enhance edges through local lateral inhibition, while higher-level cortical areas achieve an understanding of complex scenes via large-scale spatial integration. In the proposed RFSTMD model, the introduced global suppression mechanism can be regarded as a computational modeling of this hierarchical integration process; meanwhile, the feedback attention mechanism corresponds to the neural implementation of top-down attentional modulation in biological vision.

From an application perspective, the high robustness exhibited by RFSTMD makes it particularly suitable for resource-constrained embedded systems, such as micro unmanned aerial vehicles and edge-based surveillance devices. Compared with deep learning approaches, the proposed model maintains competitive detection performance while offering lower computational complexity and higher biological interpretability. Future research may explore extending this framework to multi-target tracking tasks or integrating it with other sensing modalities, such as infrared and sonar, to achieve all-weather perception capabilities.

Despite the significant progress achieved, this study still has several limitations. First, the current model is primarily designed for two-dimensional planar motion and has limited capability in perceiving depth motion in three-dimensional space. Second, the time-window length in the feedback mechanism is fixed, which may not provide optimal adaptation for targets with different velocities. Finally, the parameter λ in the global suppression mechanism requires manual tuning for specific scenarios; developing automated parameter selection strategies remains an important direction for future research. Future work can be further extended in the following directions: (1) introducing adaptive time-scale mechanisms to enable feedback modulation to dynamically adjust according to target velocity; (2) integrating stereo vision or optical-flow estimation methods to extend the model to three-dimensional motion target detection; and (3) implementing the proposed model on neuromorphic computing hardware, such as spiking neural network chips, to validate its practical value in ultra-low-power application scenarios.

## 5. Conclusions

This paper proposes a feedback-based visual neural network (RFSTMD) for detecting small moving targets in complex, dynamic environments. Leveraging significant differences in spatiotemporal response features between true targets and background artifacts, the network integrates two core pathways: a motion perception pathway for target localization and trajectory tracking, and a feedback attention pathway that extracts temporal feature envelopes to enhance selective attention to small targets. A global suppression mechanism further removes background noise. To address challenges such as extremely small target pixel ratios, low contrast, and dynamic backgrounds, the network combines feedback gain modulation with background elimination strategies, significantly improving performance. Extensive experiments on simulated and real-world datasets show that RFSTMD substantially outperforms existing benchmarks. At a false alarm rate of 3, it achieves an average detection rate of 0.81, far exceeding competing methods (all below 0.59). Meanwhile, it attains an average precision of 6.48% and an F1 score of 12%, representing orders-of-magnitude improvements over other methods. In summary, this study overcomes critical challenges in small-target detection and provides a robust, high-precision solution for visual surveillance, autonomous navigation, and bio-inspired visual systems, demonstrating substantial practical value.

## Figures and Tables

**Table 1:** Running time.

Feature	Running Time
Temporal Contrast	187.68 ms
Response Gradient	215.81 ms
Response range	89.34 ms
Standard deviation	91.00 ms

**Table 2:** Model coefficients.

Equations	Parameters
Equation (1)	$\sigma_{1}=1$, size = 3×3
Equation (3)	$n_{1}=2,\tau_{1}=3,n_{2}=6,\tau_{2}=9$, length = 2×(3+9)
Equation (7)	$n_{3}=5,\tau_{3}=25$, length = 3×25
Equation (11)	$\;\;\;\;\;\;\;\;\;\;\sigma_{2}=1.5,\sigma_{3}=3$, size = 13×13
Equation (11)	φ=1,ψ=3,e=1,ρ=0
Equation (16)	υ=35
Equation (17)	a=30
Equation (18)	λ=50
Equation (19)	$\;\;\;\;\;\sigma_{4}=15,$ size = 61×61

**Table 3:** Simulation environment and parameter settings.

Video Sequences	Original Source Video	Simulated Data 1	Simulated Data 2	Simulated Data 3	Simulated Data 4	Simulated Data 5
Object Scale Parameter	5×5	1×1∼11×11	5×5	5×5	5×5	5×5
Object Brightness Parameter	0	0	0∼0.1	0	0	0
Object Speed Parameter (pixels per second)	250	250	250	100∼300	250	250
Background Speed Parameter (pixels per second)	150	150	150	150	100∼200	100∼200
Background Direction Parameter	⟶	⟶	⟶	⟶	⟶	⟵
Background Environment	Figure 8a	Figure 8a	Figure 8a	Figure 8a	Figure 8a	Figure 8a

**Table 4:** Detection rate across various approaches ($F_{A}=3$).

Video	DSTMD	ESTMD	FSTMD	Feedback STMD	Frac-STMD	ST-STMD	RFSTMD
Original Source Video	0.72	0.79	0.89	0.54	0.13	0.81	1
Simulated one	0.35	0.47	0.52	0.45	0.15	0.46	0.76
Simulated two	0.19	0.44	0.60	0.28	0.19	0.50	1
Simulated three	0.61	0.71	0.71	0.63	0.52	0.71	1
Simulated four	0.62	0.50	0.62	0.39	0.28	0.62	0.83
Simulated five	0.43	0.55	0.55	0.50	0.25	0.58	0.67
Simulated six	0.18	0.37	0.39	0.16	0.07	0.35	0.52
Real one	0.19	0.24	0.13	0.31	0.53	0.13	0.67
Real two	0.57	0.92	0.98	0.78	0.57	0.92	1
Real three	0.16	0.31	0.36	0.41	0.36	0.34	0.63
Mean ± std	0.40 ± 0.216	0.53 ± 0.155	0.58 ± 0.179	0.45 ± 0.128	0.31 ± 0.130	0.54 ± 0.170	0.81 ± 0.131

**Table 5:** Algorithmic precision comparison.

Methods	DSTMD	ESTMD	FSTMD	Feedback STMD	Frac-STMD	ST-STMD	RFSTMD
Original Source Video	0.0083	0.0069	0.0068	0.0069	0.0083	0.0067	0.1000
Simulated one	0.0081	0.0074	0.0073	0.0067	0.0078	0.0074	0.0680
Simulated two	0.0087	0.0070	0.0070	0.0067	0.0071	0.0070	0.0930
Simulated three	0.0088	0.0069	0.0069	0.0063	0.0076	0.0070	0.1000
Simulated four	0.0109	0.0082	0.0082	0.0073	0.0149	0.0083	0.0800
Simulated five	0.0078	0.0068	0.0066	0.0061	0.0073	0.0070	0.0700
Simulated six	0.0068	0.0058	0.0058	0.0050	0.0061	0.0059	0.0400
Real one	0.0053	0.0039	0.0039	0.0041	0.0047	0.0040	0.0520
Real two	0.0079	0.0048	0.0049	0.0047	0.0058	0.0048	0.0217
Real three	0.0044	0.0037	0.0037	0.0038	0.0040	0.0037	0.0232
Mean ± std	0.008 ± 0.002	0.006 ± 0.001	0.006 ± 0.001	0.006 ± 0.001	0.0074 ± 0.002	0.0062 ± 0.001	0.0648 ± 0.021

**Table 6:** Algorithmic F1 score comparison.

Video	DSTMD	ESTMD	FSTMD	Feedback STMD	Frac-STMD	ST-STMD	RFSTMD
Original Source Video	0.016	0.014	0.014	0.014	0.016	0.013	0.18
Simulated one	0.016	0.015	0.015	0.013	0.016	0.015	0.13
Simulated two	0.017	0.014	0.014	0.013	0.014	0.014	0.17
Simulated three	0.018	0.014	0.014	0.013	0.015	0.014	0.18
Simulated four	0.020	0.016	0.016	0.014	0.029	0.016	0.14
Simulated five	0.015	0.014	0.013	0.012	0.015	0.014	0.14
Simulated six	0.013	0.016	0.012	0.010	0.012	0.012	0.08
Real one	0.011	0.008	0.008	0.008	0.009	0.008	0.10
Real two	0.016	0.010	0.010	0.008	0.012	0.010	0.04
Real three	0.009	0.007	0.007	0.008	0.008	0.007	0.04
Mean ± std	0.015 ± 0.003	0.013 ± 0.002	0.012 ± 0.002	0.011 ± 0.002	0.015 ± 0.004	0.012 ± 0.002	0.12 ± 0.038

## Data Availability

Data Availability Statement: The synthetic datasets generated during the current study and the complete source code of the RFSTMD model are publicly available in the GitHub repository at https://github.com/Alingjun920920/RFSTMD-Code-data (accessed on 10 February 2026). The real-world datasets (RIST dataset) utilized in this research can be accessed via the official repository at https://sites.google.com/view/hongxinwang-personalsite/download (accessed on 6 April 2020). Detailed execution instructions are provided within the repository to ensure full reproducibility of the presented results.
